# Chemical Profiling, Pharmacological Insights and In Silico Studies of Methanol Seed Extract of *Sterculia foetida*

**DOI:** 10.3390/plants10061135

**Published:** 2021-06-03

**Authors:** Najmul Alam, Naureen Banu, Md. Arfin Ibn Aziz, Niloy Barua, Umme Ruman, Israt Jahan, Farhana Jahan Chy, Susmita Denath, Arkajyoti Paul, Md. Nazim Uddin Chy, Mohammed Aktar Sayeed, Talha Bin Emran, Jesus Simal-Gandara

**Affiliations:** 1 Department of Pharmacy, International Islamic University Chittagong, Chittagong 4318, Bangladesh; nazmul9alam@gmail.com (N.A.); naureen2021@gmail.com (N.B.); arfinibnaziz151085@gmail.com (M.A.I.A); niloybaruaniloy@gmail.com (N.B.); umme.ruman.547@gmail.com (U.R.); istiisrat@gmail.com (I.J.); nazim107282@gmail.com (M.N.U.C.); 2 Drug Discovery, GUSTO A Research Group, Chittagong 4203, Bangladesh; arka.bgctub@gmail.com; 3 Faculty of Medicine, Rangpur Medical College, Rajshahi Medical University, Rajshahi 6000, Bangladesh; susmitasarna02@ymail.com; 4 Department of Pharmacy, BGC Trust University Bangladesh, Chittagong 4381, Bangladesh; 5 Nutrition and Bromatology Group, Department of Analytical and Food Chemistry, Faculty of Food Science and Technology, University of Vigo–Ourense Campus, E32004 Ourense, Spain

**Keywords:** *Sterculia foetida*, GC-MS, cytotoxic, thrombolytic, analgesic, antipyretic, molecular docking

## Abstract

*Sterculia foetida*, also known as jangli badam in Bangladesh, is a traditionally used plant that has pharmacological activities. A qualitative phytochemical analysis was performed to assess the metabolites in a methanolic extract of *S. foetida* seeds (MESF), and the cytotoxic, thrombolytic, anti-arthritics, analgesic, and antipyretic activities were examined using in vitro, in vivo, and in silico experiments. Quantitative studies were performed through gas chromatography-mass spectroscopy (GC-MS) analysis. The brine shrimp lethality bioassays and clot lysis were performed to investigate the cytotoxic and thrombolytic activities, respectively. The anti-arthritics activity was assessed using the albumin denaturation assay. Analgesic activity was determined using the acetic acid-induced writhing test and the formalin-induced paw-licking test. A molecular docking study was performed, and an online tool was used to perform ADME/T (absorption, distribution, metabolism, and excretion/toxicity) and PASS (Prediction of Activity Spectra for Substances). GC-MS analysis identified 29 compounds in MESF, consisting primarily of phenols, terpenoids, esters, and other organic compounds. MESF showed moderate cytotoxic activity against brine shrimp and significant thrombolytic and anti-arthritics activities compared with the relative standards. The extract also showed a dose-dependent and significant analgesic and antipyretic activities. Docking studies showed that 1-azuleneethanol, acetate returned the best scores for the tested enzymes. These findings suggested that MESF represents a potent source of thrombolytic, anti-arthritic, analgesic, antipyretic agents with moderate cytotoxic effects.

## 1. Introduction

In developing countries, medicinal plants are gaining in popularity, with 80 percent of people using it as a first therapeutic solution. A significant proportion of the world’s population (87.5%) uses herbal remedies to treat health problems. These traditional medications are typically used in the form of unrefined medications, such as tinctures, tears, poultices, powders, and other herbal formulations [1]. Medicinal plants in Bangladesh represent potential resources for a variety of factors with potential pharmacological efficiency, and more than one thousand species of flora have been documented [2]. Traditional medicinal practices remain the first course of therapy for the treatment of various illnesses, and the overwhelming majority (75–80%) of the Bangladesh population continues to desire traditional medicinal treatments due to affordability, availability, and safety, despite the increasing availability of advanced allopathic medicines [3].

*Sterculia foetida* L. is a tall tree located in Bangladesh, India, Malaysia, and North-East Australia. *S. foetida* is a tropical plant that belongs to the family Malvaceae, known as “Janglibadam” in the Hindi and Bengali languages and as “Gorapubadam” in the Tamil language [4,5]. In 1753, *S. foetida* was first depicted by Carolus Linnaeus, and is a delicate wood tree that grows up to 115 ft tall. The seeds of *S. foetida* can be eaten raw or simmered and are not harmful to humans and animals [6].

Traditionally, the leaves and seeds of *S. foetida* have been shown to display remarkable medicinal value, including as a source of sterculic acid (53%), which possesses pharmacological properties, such as anti-inflammatory and anti-nociceptive activities. The seeds of *S. foetida* are fit for human consumption, representing a good source of fats (30%–36%) and proteins (11.4%). The leaves and bark are often used as diaphoretic, diuretic, and aperient agents and have been applied to the treatment of rheumatism, obesity, gonorrhea, edema, and skin disease [7]. Based on an evaluation of the literature, this plant has been found to containing various phytoconstituents, including alkaloids, flavonoids, terpenoids, phenols, and steroids [8]. Additionally, *S. foetida* ethanolic seed extract have antioxidant and anticancer activity [6].

Although the seeds of *S. foetida* are known to have great therapeutic significance. However, no experimental and in silico evaluations have yet been reported to systematically evaluate the cytotoxic, thrombolytic, anti-arthritic, analgesic, or antipyretic properties of these seeds. Thus, in this paper, these activities were investigated.

## 2. Results

### 2.1. Qualitative Phytochemical Screening

The results of the phytochemical investigation of MESF are listed in Table S1, and the results revealed the presence of alkaloids, flavonoids, saponins, cholesterols, carbohydrates, phenols, terpenoids, steroids, and proteins in the extract.

### 2.2. GC-MS Analysis

**Figure 1:**
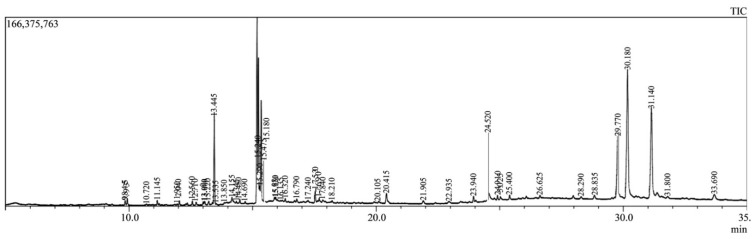
Total ionic chromatogram (TIC) of methanol extract of *Sterculia foetida* seeds by using gas chromatography-mass spectrometry (GC-MS).

Between 9.843 and 33.460 min of retention time, 29 compounds were eluted from the MESF sample (Figure 1). Comparisons with the National Institute of Standards and Technology (NIST) GC-MS library version 08-S were used to classify the compounds (Table 1). Secondary metabolites with good retention times in the methanol extract were identified as acetamiprid, halfenprox, alpha-benzene hexachloride (BHC), beta-BHC, 9-octadecenoic acid (Z), methyl ester, tralomethrin, tetradecanoic acid, methyl ester, hexadecanoic acid, methyl ester, gamma-BHC, terbufos, benfuresate, dichlofluanid, DEP (trichlorfon), sterculic acid, 1-azuleneethanol, acetate, captan, etridiazole, diethofencarb, p,p′-DDT (dichlorodiphenyltrichloroethane), etobennzanid, cyfluthrin, cypermethrin, pendimethalin, CNP (C-type natriuretic peptide), kresoxim-methyl, tetraconazole, pyributicarb, permethrin.

### 2.3. Brine Shrimp Lethality Bioassay

**Figure 2:**
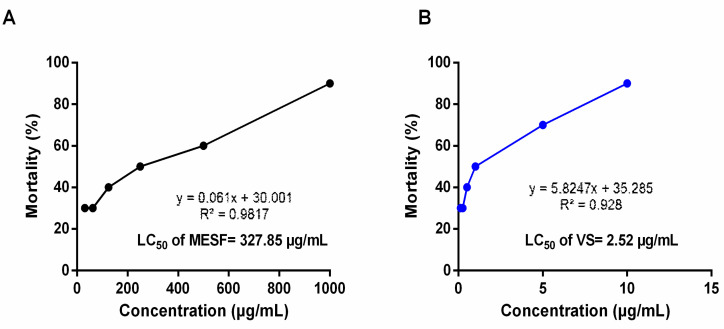
Determination of LC_50_ value of methanol extract of the *S. foetida* seeds (**A**) and standard drug, Vincristine sulfate (**B**) from linear correlation between concentrations versus percentage of mortality.

The cytotoxic effects of MESF are presented in Figure 2. The 50% lethal concentration (LC_50_) values were determined using the regression equation and the percentage of mortality following the administration of MESF at various concentrations. The LC_50_ value for MESF was determined to be 327.85 µg/mL, whereas that for vincristine sulfate was 2.52 µg/mL.

### 2.4. Thrombolytic Activity

**Figure 3:**
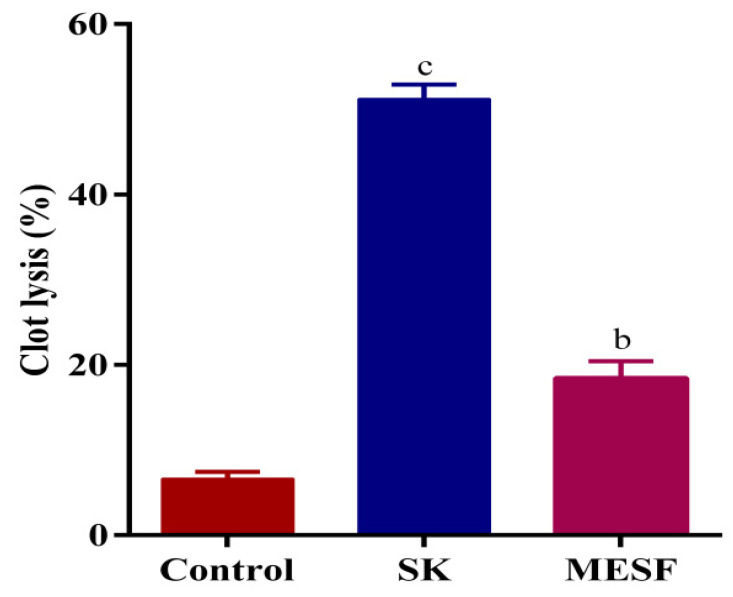
Percentage of clot lysis of human blood by MESF and standard drug. Values are represented in mean ± SEM (*n* = 3). ^b^ *p* < 0.01 and ^c^ *p* < 0.001 compared with the control group (Dunnett’s test). Here, SK: streptokinase, MESF: methanolic extract of *Sterculia foetida* seeds.

The thrombolytic activity of MESF is summarized in Figure 3. MESF displayed a higher clot lysis value (18.46% ± 1.96%) compared with the negative control of water (6.62% ± 0.83%), but a lower value than streptokinase, which was the standard drug (51.21% ± 1.68%).

### 2.5. Evaluation of In Vitro Anti-Inflammatory Activity

#### 2.5.1. Inhibition of Protein Denaturation

**Figure 4:**
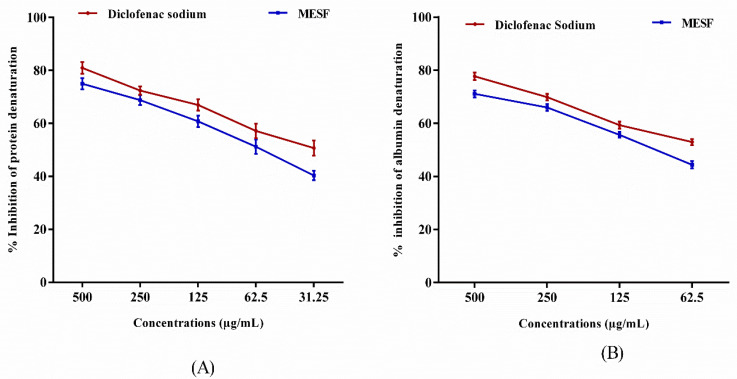
Percentage inhibition of protein (bovine serum albumin) denaturation (**A**), and albumin (egg albumin) denaturation, (**B**) comparison with using methanol extract of *S. foetida* seeds (MESF) and standard drug diclofenac sodium. Values are presented as mean ± SEM (*n* = 3).

MESF was evaluated for anti-arthritic activity using an inhibition in protein denaturation test, as shown in Figure 4A. The extract showed a dose-dependent inhibition of protein denaturation, ranging between 40.31% and 74.94% inhibition at a concentration range of 31.25–500 µg/mL. In comparison, the standard drug diclofenac sodium exhibited between 50.65% and 80.88% inhibition at a concentration range of 31.25–500 µg/mL.

#### 2.5.2. Egg Albumin Denaturation Method

The anti-arthritics potential of MESF was also evaluated by the inhibition of egg albumin denaturation, as shown in Figure 4(B). Maximum inhibition was achieved by MESF (71.08% ± 1.30%) at 500 µg/mL, whereas diclofenac sodium achieved an inhibition of 77.70% ± 1.36% at the same concentration.

### 2.6. Acute Toxicity Test

MESF, orally administered at 500–2000 mg/kg, did not result in any mortality or any visible behavioral changes in mice within the 72 h observation period following administration.

### 2.7. Analgesic Activity

#### 2.7.1. Acetic Acid-Induced Writhing Inhibition Test

**Figure 5:**
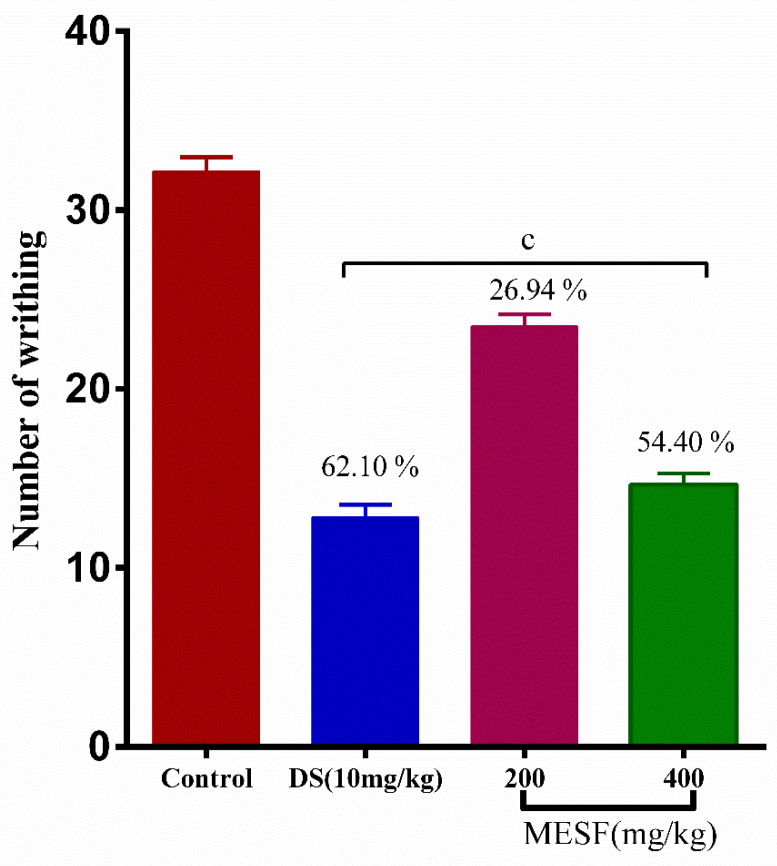
Analgesic effect of MESF in acetic acid-induced abdominal writhing test. Values are presented as mean ± SEM (*n* = 6). ^c^ *p* < 0.001 compared with the control group (Dunnett’s test). Here, DS: diclofenac sodium, MESF: methanolic extract of *Sterculia foetida* seeds.

MESF exhibited significant (*p* < 0.001) anti-nociceptive activity, as shown in Figure 5. Both tested MESF doses (200 and 400 mg/kg) inhibited writing, with 26.94% and 54.40% (*p* < 0.001) inhibition, respectively, where diclofenac sodium treatment resulted in 60.10% inhibition.

#### 2.7.2. Formalin-Induced Paw-Licking Test

**Figure 6:**
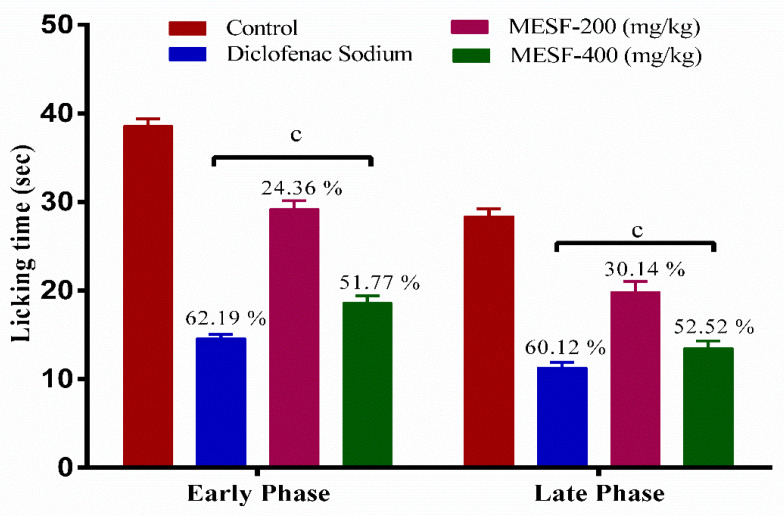
Analgesic effect of MESF in formalin-induced paw licking test. Values are presented as mean ± SEM (*n* = 6), ^c^ *p* < 0.001 compared with the control group (Dunnett’s test). Here, MESF: methanolic extract of *Sterculia foetida* seeds.

This experiment revealed that both MESF doses (200 and 400 mg/kg) resulted in the significant (*p* < 0.001) inhibition of the percentage of licking events for both the neurogenic and inflammatory phases (24.36% and 51.77%, respectively), and the result are shown in Figure 6.

#### 2.7.3. Tail Immersion Test

MESF at both 200 and 400 mg/kg (*p* < 0.01 and *p* < 0.001) significantly alleviated the observable pain induced by warm water, suggesting a notable and dose-dependent anti-nociceptive response against thermal insults. An increase in the latency times was observed for both MESF and diclofenac sodium. The percentage MPE value for both MESF and diclofenac sodium were significant (*p* < 0.01 and *p* < 0.001) at all experimental doses. However, the value for diclofenac sodium was greater than that for MESF at all observation periods for both MESF doses, as shown in Table 2.

### 2.8. Antipyretic Test by Brewer’s Yeast-Induced Pyrexia

**Figure 7:**
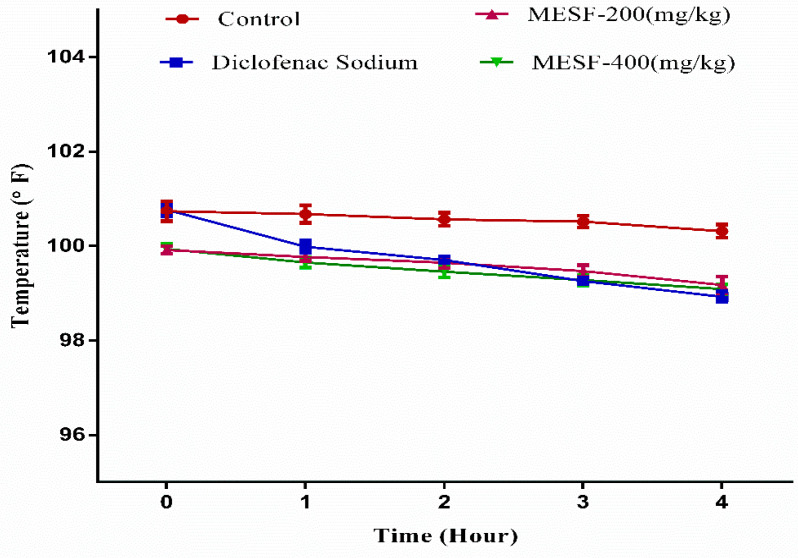
Antipyretic effect of MESF in mice. Figure presents the percent inhibition of pyrexia after 1, 2, 3, and 4 h of the treatment with diclofenac sodium and MESF (200 and 400 mg/kg). MESF: methanolic extract of *Sterculia foetida* seeds.

A significant defense against hyperthermia-induced by yeast one hour following the administration of 200 and 400 mg/kg MESF (*p* < 0.001). The antipyretic effect continued to be observed for both MESF doses 2, 3, and 4 h (*p* < 0.001) after treatment. Diclofenac sodium also significantly decreased the feverish reaction after 2 to 4 h (*p* < 0.001) of treatment and the results are shown in Figure 7.

### 2.9. Molecular Docking Related to Cytotoxic Activity

**Figure 8:**
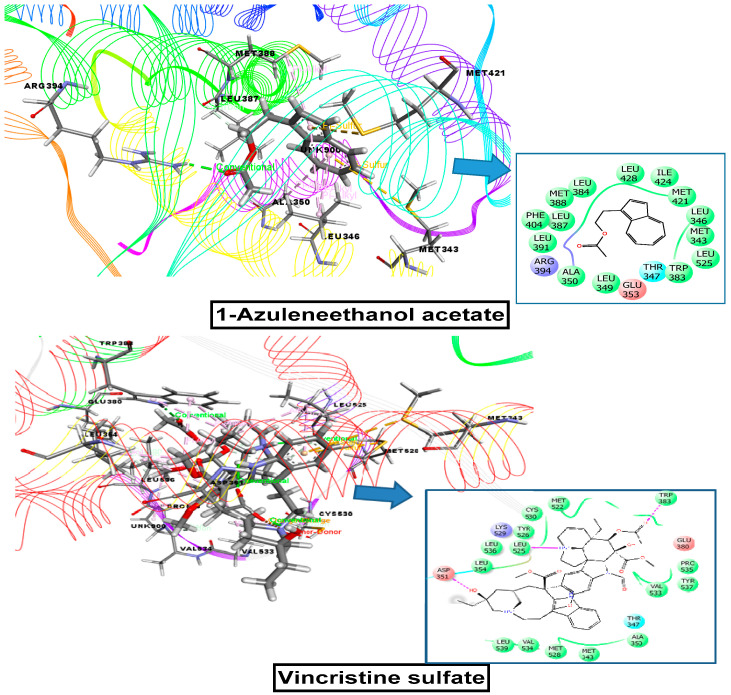
Molecular docking interaction of 1-azuleneethanol, acetate, and vincristine sulfate with human estrogen receptor for cytotoxic activity (PDB ID: 3ERT).

In this docking study, seven selected compounds found in *S. foetida* were docked with human estrogen receptor to examine for cytotoxicity activity. 1-azuleneethanol, acetate revealed the maximum interaction score of −7.215 kcal/mol binding energy with the human estrogen receptor, whereas the standard drug vincristine sulfate exhibited a binding energy of −7.059 kcal/mol for the interaction. The binding energies for the interactions between human estrogen receptor and all tested compounds are presented in Table 3 and Figure 8.

### 2.10. Molecular Docking Related to Thrombolytic Activity

**Figure 9:**
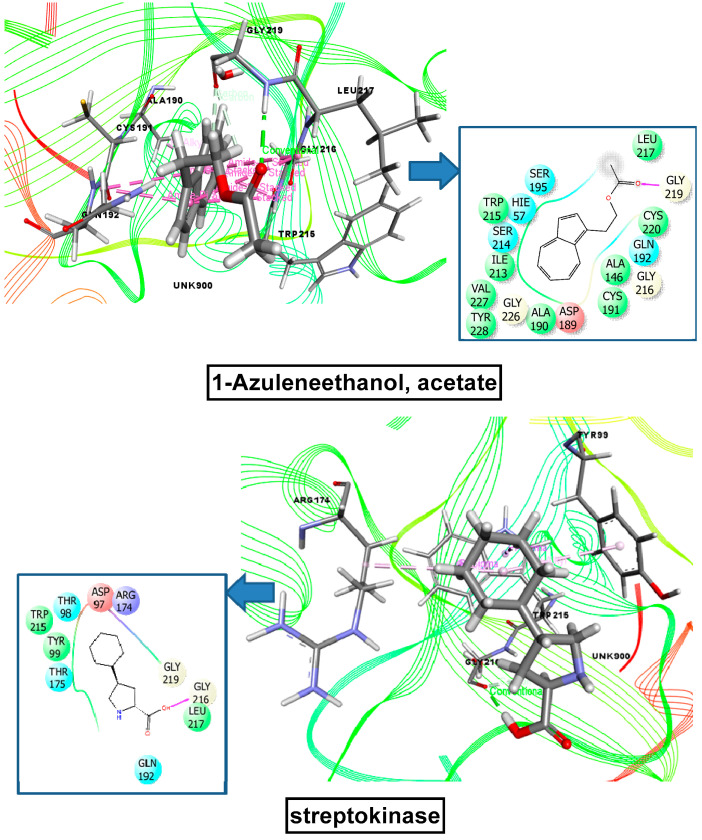
Molecular docking interaction of 1-azuleneethanol, acetate, and streptokinase with human tissue-type plasminogen activator for thrombolytic activity (PDB: 1A5H).

In this docking study, 1-azuleneethanol acetate exhibited the highest interaction score (−5.836 kcal/mol) against tissue plasminogen receptor. 1-azuleneethanol, acetate showed an even stronger binding affinity than the standard drug streptokinase (−4.533 kcal/mol). The docking scores for the tested compounds are as follows, from best to worst: 1-azuleneethanol, acetate (−7.215) > sterculic acid (−1.612) > 7, 10-octadecadienoic acid, methyl ester (−0.945) > hexadecanoic acid, methyl ester (−0.761) > tetradecanoic acid, methyl ester (0.686). 9-octadecenoic acid (Z), methyl ester did not show any interaction against tissue plasminogen receptor. All scores are shown in Table 3 and the candidate with the highest binding affinity is shown in Figure 9.

### 2.11. Molecular Docking Related to Anti-Inflammatory, Analgesic, and Antipyretic Activity

**Figure 10:**
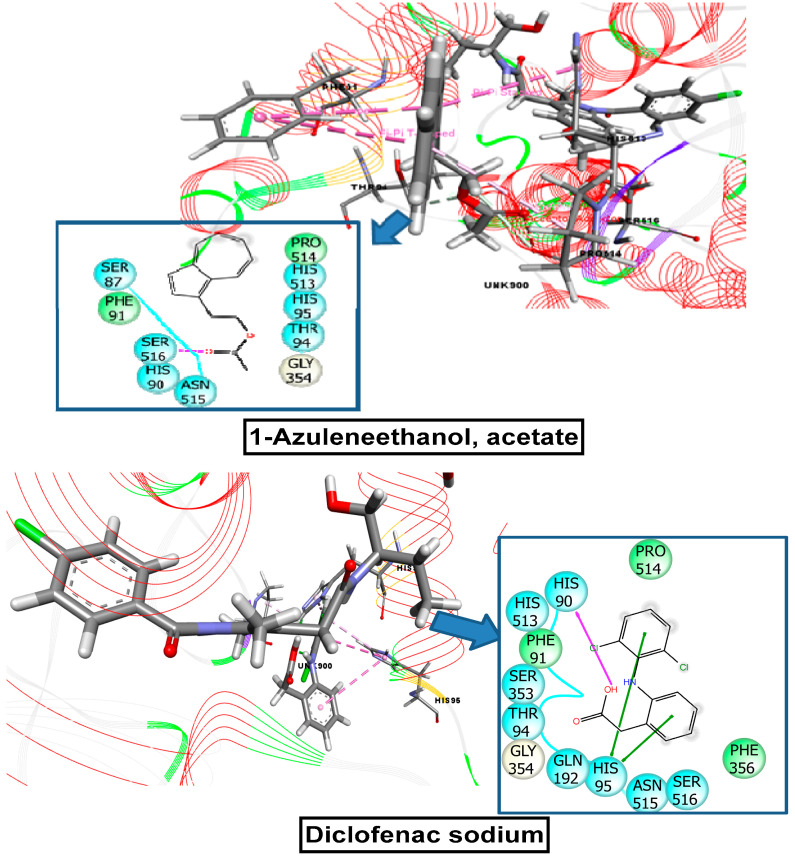
Molecular docking interaction of 1-azuleneethanol, acetate, and diclofenac-Na with COX-1 enzyme for analgesic and anti-inflammatory activity (PDB ID: 2YOE).

**Figure 11:**
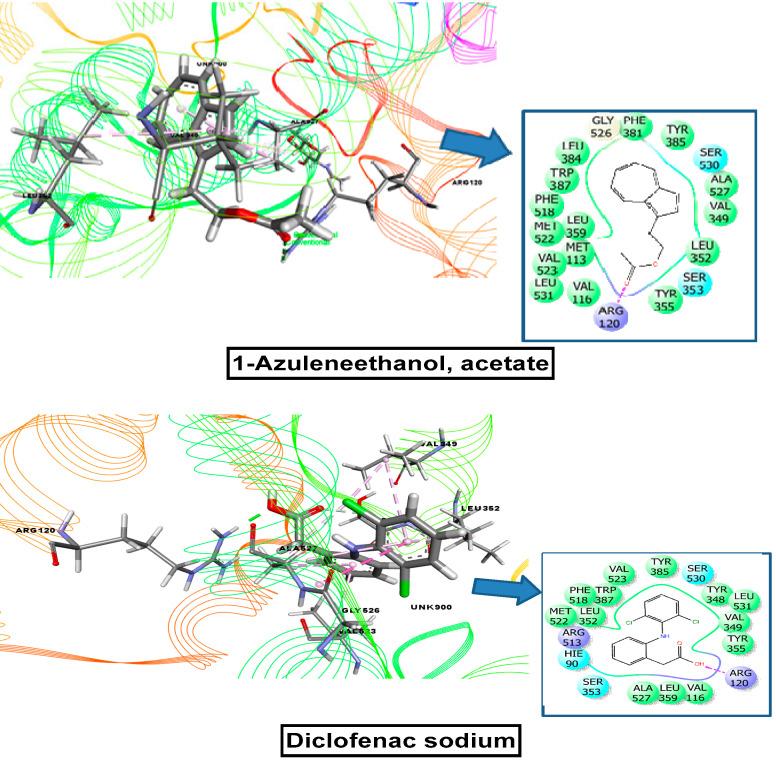
Molecular docking interaction of 1-azuleneethanol, acetate, and diclofenac-Na with COX-2 enzyme for analgesic and anti-inflammatory activity (PDB ID: 6COX).

COX-1 and COX-2 were used to investigate the anti-inflammatory, analgesic, and antipyretic potentials. For the COX-1 receptor, the strongest binding affinity was observed for 1-azuleneethanol, acetate (−5 kcal/mol), which was better than the docking score for the standard drug diclofenac sodium (−4.59 kcal/mol). The other candidates that showed docking interactions with the COX-1 receptor were as follows, in order from best to worst: 7, 10-octadecadienoic acid, methyl ester (0.42) > sterculic acid (0.525) > tetradecanoic acid, methyl ester (0.527) > hexadecanoic acid, methyl ester (0.864) > heptadecanoic acid, 14-methyl, methyl ester (2.359). In this case, 9-octadecenoic acid (Z), methyl ester did not interact with this receptor. For the COX-2 receptor, 1-azuleneethanol, acetate exhibited the best binding interaction, with a docking score of −7.602 kcal/mol, which was higher than that for the standard drug diclofenac sodium (−7.26 kcal/mol). The other compounds displayed the following binding interactions with COX-2, in order from best to worst: sterculic acid (−3.946) > 7, 10-octadecadienoic acid, methyl ester (−1.617) > tetradecanoic acid, methyl ester (−1.018) > hexadecanoic acid, methyl ester (−0.943). Neither 9-octadecenoic acid (Z), methyl ester, nor heptadecanoic acid, 14-methyl, methyl ester showed any binding affinity toward the COX-2 receptor. These scores are presented in Table 3, and binding interactions of the best-docked compounds are displayed in Figure 10 and Figure 11.

### 2.12. ADME/T and Toxicological Properties Prediction

As specified by Lipinski’s rule five, a compound may possess drug-like properties if it does not fail more than one among of the following principles: (i) MW of not more than 500 amu; (ii) HBA < 5; (iii) HBD ≤ 10 and (iv) logP ≤ 5. In addition, Veber et al. recommended that a compound should have an nRB ≤ 10 and a TPSA value ≤ 140 Å to provide optimal molecular flexibility, which might facilitate the transport of medications through membranes. Our study found that all tested compounds satisfied Lipinski’s rule of five and Veber’s rule, which indicated that each compound could represent a good starting point for the development of new drugs (Table 4). The toxic properties of the compounds were also predicted by the admetSAR online tool and the results are shown in Table 5. All of the tested compounds showed Ames toxicity, carcinogenicity, acute oral, and rat acute toxicity properties.

### 2.13. PASS Prediction Study

The seven selected compounds found in MESF were studied using the PASS online tool to assess cytotoxic, thrombolytic, anti-inflammatory, anti-nociceptive, antipyretic, and other biological activities. Potent molecules displayed higher Pa values than Pi values. This study identified a number of essential biological activities for each compound, including anti-inflammatory, anti-eczematic, anti-secretory, antithrombotic, anti-nociceptive, antipyretic, anti-hypoxic, anti-seborrheic, hypolipemic, antipruritic, and acetyl esterase inhibiting activities (Table 6).

## 3. Discussion

Plants synthesize phytoconstituents known as secondary metabolites, which are not essential for plant growth or survival and the metabolites of plants have often been found to provide medical benefits to humans [9]. In previous studies, a methanol extract of *S. foetida* seed powder identified a total of 13 compounds, including phenols, flavonoids, esters, and other organic compounds [10]. In contrast, another study of ethanolic extract of *S. foetida* seed powder revealed 35 bioactive compounds [6]. However, the phytochemical analysis performed for the plant-derived MESF revealed the existence of various phytochemicals, such as alkaloids, flavonoids, saponins, cholesterols, carbohydrates, phenols, terpenoids, steroids, and proteins, which were examined based on the literature review [8]. In addition, no deaths or unusual behaviors were observed in mice treated with doses of up to 2000 mg/kg MESF during the acute toxicity studies, indicating that the extract has a low-toxicity profile. The present study aimed to perform a qualitative examination of the presence of various types of phytochemicals in MESF and then examined the efficiency of MESF as a cytotoxic, thrombolytic, anti-inflammatory, anti-nociceptive, and antipyretic agent.

The brine shrimp lethality assay is commonly used to examine cytotoxicity and can also be used to examine a broader array of pharmacological activities, including the antibacterial, pesticidal, antiviral, and antitumor activities of plant extracts and compounds [11]. Multiple concentrations of MESF, ranging from 31.5 to 1000 mg/mL, were tested to examine the cytotoxic effects of MESF. The cytotoxicity is shown in Figure 1, and the crude methanol extract displayed a moderate cytotoxic effect according to the determined LC₅₀ value, which signifies the protective effects of the extract at therapeutic doses [12]. In addition, the cytotoxic effects of the plant components and secondary metabolites depend on the interactions among these factors, which is consistent with our current investigation and previous investigations, in which both qualitative and quantitative assessments revealed the presence of alkaloids, flavonoids, and terpenoids, which may have anticancer properties [13,14].

Various studies have been conducted to determine whether herbs, natural food sources, and supplements display thrombotic effects, which have been authenticated by the ingestion of these types of foods for the prevention of stroke and coronary-associated adverse outcomes [15,16,17,18,19]. The results of the thrombolytic bioassay suggested that the extract showed significant thrombolytic activity, indicating that *S. foetida* should be evaluated through further research for thrombolytic activity against various diseases.

Inflammation is defined as a complex pharmacological response of body tissues to certain harmful stimuli such as pathogens, damaged cells, etc. which leads to the formation of redness, swelling, and ultimately the pain. Additionally, activated macrophages yield numerous pro-inflammatory cytokines and subsequently stimulate cellular feedback which is primarily responsible for the formation of two major types of inflammations, viz. acute and chronic inflammations. In this test, protein denaturation can occur due to the disruption of various electrostatic, hydrogen, hydrophobic, and disulfide bonds and heat-denatured proteins have been found to be more effective than all-natural proteins for the inducement of delayed hypersensitivity [20,21]. Furthermore, it has been established that conventional NSAIDs such as phenylbutazone and indomethazine act not only by inhibiting the development of endogenous prostaglandins by inhibiting the COX enzyme but also by preventing protein denaturation [22]. In the current study, the plant extract was presented a high level of inhibitory activity against protein denaturation, which was similar to that observed for the standard drug diclofenac sodium. The maximal ability to repress protein denaturation indicated that *S. foetida* extract could protect against thermal protein denaturation. Secondary metabolites detected in preliminary phytochemicals, such as saponins and terpenoids may be responsible for this activity.

Traditional practitioners investigate the therapeutic uses of plants based on pharmacological actions, which represent a fundamental part of ethnopharmacology [23]. New anti-nociceptive agents derived from plants are continuously being discovered based on existing ethnopharmacology knowledge [24]. We first examined the anti-nociceptive activity of MESF using the acetic acid-induced writhing test. The intraperitoneal administration of acetic acid triggers the release of endogenous substances, such as cytokinins, bradykinin, histamine, cyclooxygenase, serotonin, and prostaglandin, which enter the dorsal horn of the central nervous system, acting as visceral inflammatory mediators to trigger the activation of primary afferent nociceptors and induce pain, which presents as abdominal constriction [25,26,27]. In our experiment, the writhing of mice decreased notably in response to the oral administration of MESF, resulting in significant anti-nociceptive activity.

Both the central and peripheral anti-nociceptive activities of MESF were examined, and the formalin test was performed to differentiate between these two types of activity. Chemical nociceptors, especially C-fibers, directly stimulate sensory afferents to mediate formalin-induced nociception during the early phase, inducing the production of peripheral inflammatory mediators, such as bradykinin, serotonin, histamine, and prostaglandin during the late phase [28,29]. Both phases possess their own attributes, which can be employed as a tool to explore the mechanisms of anti-nociception and assess the anti-nociceptive effect. Narcotics and opioid drugs, including morphine and heroin, act centrally to suppress both phases of the formalin test, whereas non-steroidal anti-inflammatory drugs (NSAIDs), such as diclofenac sodium, indomethacin, aspirin, and dexamethasone, act peripherally and suppress only the late phase [30]. MESF resulted in anti-nociceptive effects against both the neurogenic and inflammatory phases of the formalin-induced paw-licking test, which implied that the extract acts through both central and peripheral anti-nociceptive effects. These effects be mediated by the presence of flavonoids, alkaloids, and terpenoids, the bioactive compounds found in MESF that are known to exert analgesic and anti-inflammatory activities [31,32,33,34].

We performed the tail immersion test to further examine the central anti-nociceptive activity of MESF. The response inhibition and elevated latency times observed during this test were significant, suggesting the specific central analgesic activity of MESF. The response of the tail immersion test has been determined to selectively identify central analgesia, whereas peripherally acting agents have no effects on thermal stimuli [35]. Nociception is induced via spinal reflexes, and the response to the tail immersion test is mediated by µ2, κ1, and δ2 opioid receptors [36,37]. The anti-nociceptive activity of MESF in the tail immersion test indicates that MESF may affect spinal and supra-spinal receptors.

Many exogenous pyrogens in animal models, including bacterial endotoxins and microbe infections can induce fever, and are responsible for the production of various proinflammatory cytokines. These cytokines can stimulate the local release of prostaglandins into the hypothalamic circulation, shifting the hypothalamic set point and modulating body temperature to maintain a fine balance between the production and loss of heat. The hypothalamus maintains body temperature and regulates the setpoint, and NSAIDs can suppress the synthesis of prostaglandins in the hypothalamus to exert their antipyretic actions [34,38]. The present study found that MESF showed remarkable antipyretic effects in a yeast-induced model, which resulted in the elevation of body temperature. MESF had a similar effect on body temperature as the standard drug, diclofenac sodium. The extract may also apply its antipyretic action within the hypothalamus through the inhibition of prostaglandin synthesis, resulting in antipyretic action similar to NSAIDs. Although no evidence has been provided to suggest the ability of *S. foetida* to disrupt prostaglandin synthesis in the hypothalamus [39,40], the current pharmacological findings support the claims made by traditional medicinal practitioners that *S. foetida* seeds can act as an antipyretic agent.

Computational molecular docking studies represent a vital method for determining the potential binding capabilities of active biological constituents against selected proteins [41]. Seven compounds were docked against four target receptors and among all the examined compounds, 1-azuleneethanol, acetate showed a more favorable binding affinity toward each receptor than the reference drugs. In addition, the ADME/T analysis demonstrated that 1-azuleneethanol, acetate meets Lipinski’s rule of five and does not possess any toxic properties, including Ames toxicity, carcinogenicity, acute oral, or rat acute toxicity (Table 5 and Table 6). Accordingly, 1-azuleneethanol, acetate can be considered a promising drug candidate, with good oral bioavailability and multiple pharmacological actions. However, subsequent study remains necessary to isolate the compounds in a pure form to better understand the molecular mechanism and to ensure the long-term safety of compound use. In addition, the significant results demonstrating the thrombolytic, anti-inflammatory, analgesic, and antipyretic potentials of MESF may conceivably be associated with the presence of 1-azulenethanol, acetate.

The seven chemical constituents from *S. foetida* seeds were subjected to a docking study using the human estrogen receptor, tissue plasminogen receptor, cyclooxygenase-1, cyclooxygenase-2 as receptors. The docking analysis showed that 1-azuleneethanol, acetate presented the highest binding affinity with all four receptors: 3ERT (−7.215 kcal/mol), 1A5H (−5.836 kcal/mol), 2OYE (−5 kcal/mol), and 6COX (−7.602 kcal/mol) activity. These docking scores were higher than those for the standard drugs: vincristine sulfate (−7.059 kcal/mol) as a cytotoxic drug; streptokinase (−4.533 kcal/mol) as a thrombolytic agent; and diclofenac sodium (−4.59 and −7.26 kcal/mol) as an anti-arthritic, analgesic and antipyretic.

## 4. Materials and Methods

### 4.1. Plant Collection and Extraction Process

The seeds of *S. foetida* were collected from Barkup, Kutubdia, Cox’s Bazar, Bangladesh in May 2019 and were identified by Professor Shaikh Bokhtear Uddin, Department of Botany, University of Chittagong, Chittagong, Bangladesh. A voucher sample was deposited within the herbarium for additional reference (DACB: 35459) [4]. The methanol extract of *S. foetida* (MESF) was prepared by macerating ground dried seeds (800 g) in methanol (2 L) with random stirring for 15 days. The solution was evaporated to dryness in a rotary evaporator obtaining a 20 g extract.

### 4.2. Chemicals

The following analytical-grade chemical reagents were used in all investigations; methanol, purchased from Merck, Darmstadt, Germany, bovine serum albumin (BSA) (D Fine Chem., Ltd., Mumbai, India), and lyophilized streptokinase vial (1,500,000 IU) (Beacon Pharmaceuticals Bangladesh, Ltd., Dhaka, Bangladesh), diclofenac sodium (Square Pharmaceuticals Ltd., Dhaka, Bangladesh), acetic acid, and formalin (Merck, Mumbai, India). All other chemicals were purchased from Taj Scientific, Ltd. (Chittagong, Bangladesh).

### 4.3. Experimental Animals and Ethical Statement

Mice (Swiss albino), weighing 20–30 g, were obtained from the International Center for Diarrheal Diseases Research, Dhaka, Bangladesh (ICDDR,B). The experimental animals were provided with access to regular laboratory food and potable water and were maintained on a day-light cycle in a room with suitable air circulation. All animal experiments were performed in a separate room under soundless conditions. All animal experiments were performed at the Department Pharmacy, International Islamic University Chittagong, Chittagong, Bangladesh. All mice were acclimated to laboratory conditions for 14 days before starting experiments. For all animal experiments, all efforts were made to minimize the suffering of the animals. At the end of the observation period, all mice were euthanized using diethyl ether anesthesia. All protocols associated with this experiment were approved by the “P&D committee” Department of Pharmacy, International Islamic University Chittagong, Chittagong, Bangladesh, according to current government guidelines (under the reference number, Pharm/P&D/151/24–2019). All sections of this report adhere to the Animal Research: Reporting of In Vivo Experiments (ARRIVE) guidelines for the reporting of animal research. “Principles of the Laboratory Animal Care” (NIH publication no. 85–23, revised 1985) and “National Animal Care Laws” were strictly followed during the handling of all animals during this study.

### 4.4. Qualitative Phytochemicals Analysis

The MESF was qualitatively screened for the presence of bioactive compounds using standard procedures to evaluate the presence of alkaloids, glycosides, quinones, flavonoids, saponins, cholesterols, carbohydrates, phenols, terpenoids, steroids, and proteins [42,43].

### 4.5. GC-MS (Gas Chromatography-Mass Spectrometry) Analysis

The MESF was analyzed in a mass spectrometer (TQ 8040, Shimadzu Corporation, Kyoto, Japan) using the electron impact ionization (EI) method and a gas chromatograph (GC-17A, Shimadzu Corporation) with a fused silica capillary column (Rxi-5 ms; 0.25 m film, 30 m long and internal diameter 0.32 mm) coated with DB-1 (J&W). The oven temperature was set at 70 °C (0 min); 10 °C, 150 °C (5 min); 12 C, 200 °C (15 min); 12 °C, 220 °C (5 min), with a hold time of 10 min. The inlet temperature was 260 °C. The flow rate of the column was 0.6 mL/min helium gas at constant pressure (90 kPa). The GC to MS interface temperature was 280 °C. The MS was used in scanning mode, with a scanning range of 40–350 amu. The ionization mode was electron ionization (EI), and the mass range was 50–550 m/z. One microliter of the sample was injected in the split-less injection mode. The total GC-MS run time was 50 min. The compounds in the peak areas were identified by comparison with the national institute of standards and technology (NIST) GC-MS library version 08-S. 

### 4.6. Brine Shrimp Lethality Bioassay

The cytotoxicity of MESF was examined against brine shrimp (*Artemia salina*), using a previously established method [44]. Briefly, 1 L distilled water (DW) was combined with 38 g sodium chloride to prepare artificial seawater, and MESF was serially diluted to obtain concentrations of 31.25–1000 µg/mL. Vincristine sulfate (the standard drug) was used at serially diluted concentrations (0.125, 0.25, 0.5, 1, 5, and 10 µg/mL) was used as the positive control. Ten living brine shrimp were applied to each vial. After 24 h, each vial was examined with a magnifying glass, and the living shrimp were counted and recorded.

### 4.7. Thrombolytic Activity

The thrombolytic activity of MESF was evaluated by utilizing a previously described method, with minor modifications [45,46], using streptokinase as the standard. A streptokinase vial (1,500,000 IU) was obtained commercially and combined with 5 mL DW to generate a stock solution. A 100 µL streptokinase dose from this stock solution was utilized for the in vitro investigation. Blood was obtained from five healthy students, excluding any participants with any history of antithrombotic treatment. The collected blood was aliquoted into a pre-weighed microcentrifuge tube to allow for clot development, as described in our previous research [47]. The difference in the weight before and after the clot lysis assay was used to determine the percentage of clot lysis, according to the following formula:

% clot lysis = (weight of the clot after removing fluid/initial weight of the clot) × 100%

This human-related experiment was conducted according to the standards established by the 1964 Declaration of Helsinki. This study protocol was approved by the Department of Pharmacy, International Islamic University Chittagong, Chittagong, Bangladesh (ref. number: Pharm/P&D/151/24–2019).

### 4.8. Evaluation of In Vitro Anti-Inflammatory Activity

#### 4.8.1. Inhibition of Protein Denaturation

A previously described method, with minor modifications, was used to examine the inhibition of protein denaturation to determine the anti-arthritic activity of MESF [48,49]. The 0.5 mL test sample contained 0.05 mL MESF extract at serially diluted concentrations from 31.25–500 µg/mL and 0.45 mL BSA. The 0.5 mL test control solutions contained 0.45 mL BSA and 0.05 mL of either DW (negative control) or diclofenac sodium (pH 6.3, positive control). The samples were incubated at 37 °C for 20 min and then at 57 °C for 30 min. Phosphate-buffered saline (PBS, 2.5 mL) was added, and absorbance was measured at 660 nm. The inhibition of protein denaturation was calculated as follows:

$$\%\;\mathrm{Inhibition}=\left[\frac{\mathrm{Ac}-\mathrm{As}}{\mathrm{Ac}}\right]\times 100$$

where Ac = absorbance of the control and As = absorbance of the sample.

#### 4.8.2. Egg Albumin Denaturation Method

The anti-arthritic activity of the extracts was determined using the previously established technique, with minor modifications [50]. An approximately 5 mL mixture, containing 0.2 mL egg albumin (hen’s egg, 0.2 mL), 2.8 mL PBS (pH 6.4) and variable concentrations of MESF (2 mL) were combined to obtain a final extract concentration of 62.5, 125, 250, and 500 µg/mL of the extract. The reference drug diclofenac sodium was used as a positive control and distilled water (DW) was the negative control. The mixture was incubated for 15 min at 37 ± 2 °C, followed by incubation for 5 min at 70 °C. Using the vehicle as a blank, the absorbance was obtained at 660 nm. The inhibition of albumin denaturation was calculated using the following formula:

$$\%\;\mathrm{Inhibition}=\frac{\mathrm{Ac}-\mathrm{At}}{\mathrm{Ac}}\times 100$$

where Ac = absorbance of the control and At = absorbance of the tested sample.

### 4.9. Acute Toxicity Test

The acute oral toxicity test was completed according to the guidelines established for the Organization for Environmental Control Development (OECD: guidelines 420; fixed-dose method). Animals were randomly separated into groups (*n* = 6) that received varying doses of MESF (up to 2000 mg/kg) by oral administration. The control group received water. The animals were observed for 72 h for any behavioral changes, allergic reactions, or mortality [51].

### 4.10. Analgesic Activity

#### 4.10.1. Acetic Acid-Induced Writhing Inhibition Test

Analgesic activity was tested using an acetic acid writhing inhibition method, as previously described, and the mice were separated into four groups (*n* = 6). Groups I, II, III, and IV mice were orally administered Tween-80 (10 mL/kg), diclofenac sodium (10 mg/kg), and MESF (200 mg/kg and 400 mg/kg) consistently. Thirty minutes following the drug treatment administration, 0.7% acetic acid was administered intraperitoneally, and the writhing time was measured, starting five minutes after acetic acid administration and lasting for a period of 20 min [52,53]. At the end of the experiment, the mice were euthanized using diethyl ether anesthesia.

#### 4.10.2. Formalin-Induced Paw-Licking Test

Animals were randomly divided and treated as described for the acetic acid-induced writhing test. After 30 min of drug administrations, 2.5% formalin solution was injected subcutaneously into the hind paws of all mice, and the licking behaviors were recorded from 0–5 min, as the early phase, and from 15–30 min, as the late phase [54]. At the end of the experiment, the mice were euthanized using diethyl ether anesthesia.

#### 4.10.3. Tail Immersion Test

The tail immersion test was performed according to a previously established method to estimate the central analgesic properties of MESF. The animals were randomly divided and treated as described for the acetic acid-induced writhing test. After drug administration, 2–3 cm of the tail from each mouse was placed in warm water at a constant temperature of 55 ± 0.5 °C, and the abrupt removal of the tail from the warm water is considered to represent a pain reaction. Afterward, the total tail immersion time and tail deflection at zero, thirty, sixty, ninety, and one hundred twenty minutes after the administration of all test samples were noted as response times; to inhibit tail tissue impairment in mice, a cutoff period of 20 s was used for the placement of the tail in warm water [55]. At the end of the experiment, the mice were euthanized using diethyl ether anesthesia. The percentage maximum possible effect (%MPE) for individual mice was measured according to the following formula:

%MPE=[(posttreatment latency−pretreatment latency)/(cut of time−pretreatment latency)]×100

### 4.11. Antipyretic Activity Assay Using Brewer’s Yeast-Induced Pyrexia

Mice were randomly separated into four treatment groups (*n* = 6), and all animals were fasted overnight, with free access to drinking water. The regular rectal temperature of every mouse was noted via a digital thermometer, and a 20% aqueous suspension of brewer’s yeast (10 mL/kg, subcutaneously; s.c.) was administered to all mice to induce pyrexia, and after 24 h, the rectal temperature of each mouse was documented, and the induction of pyrexia was confirmed by a rising temperature of more than 0.5 °C [53]. Briefly, the mice Groups I, II, III, and IV were orally treated once with Tween-80 (10 mL/kg), diclofenac sodium (10 mg/kg), or MESF (200 mg/kg and 400 mg/kg). The temperature of each animal was periodically recorded at 1, 2, 3, and 4 h after drug administration, and the activity of each treatment was evaluated. At the end of the experiment, the mice were euthanized using diethyl ether anesthesia.

### 4.12. Compounds Selection for the Computational Analysis

1-azuleneethanol, acetate (PubChem CID: 588184), 7,10-octadecadienoic acid, methyl ester (PubChem CID: 549028), 9-octadecenoic acid (Z), methyl ester (PubChem CID: 5354176), heptadecanoic acid, 14-methyl, methyl ester (PubChem CID:17219), hexadecanoic, methyl ester (PubChem CID:8181), sterculic acid (PubChem CID: 12921), and tetradecanoic acid, methyl ester (PubChem CID: 31284) were selected based on the GC-MS analysis and literature review [56]. The chemical structures were downloaded from the PubChem database.

### 4.13. Molecular Docking Analysis

In this docking study, the Ligprep tool, and protein preparation wizard were utilized for the preparation of the ligand and proteins, respectively, and both were fixed in Schrödinger-Maestro v 10.1. The following 3D crystallographic structures were obtained from the Research Collaboratory for Structural Bioinformatics Protein Data Bank (RCSB PDB) [57]: human estrogen receptor for cytotoxic activity (PDB ID: 3ERT) [58], human tissue-type plasminogen activator for thrombolytic activity (PDB: 1A5H) [59], and cyclooxygenase-1 (COX-1, PDB ID: 2OYE) [60] and cyclooxygenase-2 (COX-2, PDB ID: 6COX) [61] for analgesic and anti-inflammatory activities.

### 4.14. ADME Analysis and Toxicological Property Prediction

The pharmacokinetic properties (absorption, distribution, metabolism, and excretion, ADME) of the compounds were determined using Swiss ADME (http://www.swissadme.ch/) (accessed on 2 October 2020). Molecular descriptors, including molecular weight (MW), hydrogen bond acceptor (HBA), hydrogen bond donor (HBD), lipophilicity (LogP), atom molar refractivity (AMR), number of rotatable bonds (nRB), topological polar surface area (TPSA), and violations of Lipinski’s rule of five were calculated because orally active medications should comply with these widely used drug-likeness properties to establish their medicinal credibility. In addition, the toxic properties of the complexes were predicted by the admetSAR online server (http://lmmd.ecust.edu.cn/admetsar2//) (accessed on 2 October 2020), as toxicity is an important issue during drug development.

### 4.15. PASS Prediction

The seven major phytoconstituents, 1-azuleneethanol, acetate; 7, 10-octadecadienoic acid, methyl ester; 9-octadecenoic acid (Z), methyl ester; heptadecanoic acid, 14-methyl, methyl ester; hexadecanoic acid, methyl ester; sterculic acid; tetradecanoic acid, methyl ester, were investigated to estimate the antithrombotic, anti-inflammatory, anti-nociceptive, antipyretic, and other biological activities and those with potent activities exhibited higher active probability (Pa) value than inactive probability (Pi) values which assessed by the PASS online program (http://www.pharmaexpert.ru/passonline/predict.php) (accessed on 2 October 2020).

### 4.16. Statistical Analysis

All values are reported as the mean ± SEM (standard error mean). Dunnett’s test was applied to compare values, as appropriate, using SPSS v 20.0 software. Values were considered significant at *p* < 0.05, 0.01 and 0.001.

## 5. Conclusions

This study suggested that MESF possesses significant thrombolytic, anti-arthritic, analgesic, antipyretic activities with a moderate cytotoxic effect. These activities could be due to the synergistic effects of secondary metabolites found abundantly in this extract. Our computational study revealed that 1-azuleneethanol, acetate has a higher binding affinity with the human estrogen receptor, involved in cytotoxic effects; tissue plasminogen receptor involved thrombolytic effects; cyclooxygenase-1, and cyclooxygenase-2, involved in anti-arthritic, analgesic, and antipyretic effects. Additionally, the ADME characteristics, toxicological properties, and PASS were assessed for all compounds. These findings suggest that an overall 1-azuleneethanol acetate could be beneficial for the subsequent development of clinical applications. However, the additional, comprehensive analysis of the extract and associated fractions, followed by the isolation and identification of significant isolates, is strongly recommended to confirm which bioactive isolates are responsible for the observed pharmacological properties. In-depth studies suggested elucidating the possible mechanisms of animal models and humans to verify clinical efficacy.

## Figures and Tables

**Table 1:** Quantitative compounds identified from methanol extract of *Sterculia foetida* by GC-MS analysis.

SL. No.	Compound Name	Retention Time	Chemical Formula
1.	Acetamiprid	9.843, 12.039, 13.450, 14.484 and 15.882	C_10_H_11_ClN_4_
2.	Halfenprox	9.843, 12.713, 15.179, 17.529 and 17.841	C_24_H_23_BrF_2_O_3_
3.	Alpha-BHC	9.843, 13.450, 13.450, 14.161, 15.179, 17.836, 20.417 and 24.525	C_6_H_6_Cl_6_
4.	Delta-BHC	9.843, 11.143, 12.713 and 15.882	C_6_H_6_Cl_6_
5.	Beta-BHC	9.843	C_6_H_6_Cl_6_
6.	9-octadecenoic acid (Z), methyl ester	9.843	C_21_H_38_O_4_
7.	Tralomethrin	9.927, 12.562, 15.178, 15.178, 16.789 and 29.780	C_22_H_19_Br_4_NO_3_
8.	Tetradecanoic acid, methyl ester	11.143	C_15_H_30_O_2_
9.	Hexadecanoic acid, methyl ester	11.143	C_17_H_34_O_2_
10.	Gamma-BHC	12.039, 13.450, 15.179, 20.416 and 22.935	C_6_H_6_Cl_6_
11.	Terbufos	12.039, 15.477 and 17.529	C_9_H_21_O_2_PS_3_
12.	Benfuresate	12.039, 13.450, 14.483, 16.321, 17.530 and 31.146	C_12_H_16_O_4_S
13.	Dichlofluanid	13.450, 14.162, 17.529, 17.530 and 22.935,	C_9_H_11_Cl_2_FN_2_O_2_S_2_
14.	DEP (Trichlorfon)	13.449, 15.179, 15.882 and 20.417	C_4_H_8_Cl_3_O_4_P
15.	Sterculic acid	14.483	C_19_H_34_O_2_
16.	1-azuleneethanol, acetate	14.484	C_14_H_14_O_2_
17.	Captan	15.178	C_9_H_8_Cl_3_NO_2_S
18.	Etridiazole	15.882	C_5_H_5_Cl_3_N_2_OS
19.	Diethofencarb	17.841 and 28.319	C_14_H_21_NO_4_
20.	p, p′-DDT	21.907	C_14_H_9_Cl_5_
21.	Etobennzanid	23.940	
22.	Cyfluthrin	24.526 and 25.404	C_22_H_18_Cl_2_FNO_3_
23.	Cypermethrin	24.526 and 25.404	C_22_H_19_Cl_2_NO_3_
24.	Pendimethalin	26.624	C_13_H_19_N_3_O_4_
25.	CNP	28.932	C_93_H_157_N_27_O_28_S_3_
26.	Kresoxim-methyl	29.780 and 30.179	C_18_H_19_NO_4_
27.	Tetraconazole	29.780 and 30.179	C_13_H_11_Cl_2_F_4_N_3_O
28.	Pyributicarb	29.780 and 30.179	C_18_H_22_N_2_O_2_S
29.	Permethrin	25.404, 26.624, 32.125 and 33.460	C_21_H_20_Cl_2_O_3_

**Table 2:** Analgesic effect of MESF in the tail immersion test.

Treatment	Dose (mg/kg)	Response Times (s) (% MPE)				
		Pretreatment	30 min	60 min	90 min	120 min
**Control**		3.24 ± 0.26	3.95 ± 0.12	3.62 ± 0.12	3.25 ± 0.23	2.89 ± 0.14
**Diclofenac** **sodium**	10	3.72 ± 0.11	6.67 ± 0.32 ^c^ (18.10)	8.66 ± 0.25 ^c^ (30.32)	7.33 ± 0.21 ^c^ (22.20)	6.88 ± 0.26 ^c^ (19.37)
**MESF**	200	3.57 ± 0.24	5.02 ± 0.16 ^b^ (8.81)	5.44 ± 0.08 ^c^ (11.40)	5.89 ± 0.16 ^c^ (14.14)	5.42 ± 0.14 ^c^ (11.27)
	400	3.30 ± 0.08	5.82 ± 0.18 ^c^ (15.14)	6.83 ± 0.12 ^c^ (21.14)	6.42 ± 0.11 ^c^ (18.70)	6.0 9± 0.12 ^c^ (16.72)

Values are mean ± SEM (𝑛 = 6). ^b^ *p* < 0.01 and ^c^ *p* < 0.001 represented highly significant compared to control (Dunnett’s test). Here, MESF: methanolic extract of *Sterculia foetida* seeds.

**Table 3:** Molecular docking analysis of major bioactive compounds.

Compounds Name	CID Number	Docking Score (kcal/mol)			
		Cytotoxic (3ERT)	Thrombolytic (1A5H)	COX1 (2OYE)	COX2 (6COX)
1-azuleneethanol, acetate	588184	−7.215	−5.836	−5.000	−7.602
7,10-octadecadienoic acid, methyl ester	549028	−2.482	−0.945	0.420	−1.617
9-octadecenoic acid (Z), methyl ester	5354176	-	-	-	-
Heptadecanoic acid, 14-methyl, methyl ester	17219	-	1.04	2.359	-
Hexadecanoic acid, methyl ester	8181	−0.166	−0.761	0.864	−0.943
Sterculic acid	12921	−2.759	−1.612	0.525	−3.946
Tetradecanoic acid, methyl ester	31284	−0.055	0.686	0.527	−1.018
Standard drug (Vincristine sulfate, Streptokinase, Diclofenac sodium)	−7.059	−4.533	−4.590	−7.260

**Table 4:** Physicochemical properties of the compounds for good oral bioavailability.

Compounds	MW	HBA	HBD	LogP	AMR	nRB	TPSA	Lipinski’s Violations
Rules	<500	<5	≤10	≤5	40–130	≤10	≤140	≤1
1-azuleneethanol, acetate	214.26 g/mol	2	0	2.95	63.74	4	26.30 Å^2^	0
7, 10-octadecadienoic acid, methyl ester	294.47 g/mol	2	0	5.68	93.78	15	26.30 Å^2^	1
9-octadecenoic acid (Z), methyl ester	354.52 g/mol	4	0	5.74	105.16	18	52.60 Å^2^	1
Heptadecanoic acid, 14-methyl, methyl ester	298.50 g/mol	2	0	6.21	94.73	16	26.30 Å^2^	1
Hexadecanoic acid, methyl ester	270.45 g/mol	2	0	5.54	85.12	15	26.30 Å^2^	1
Sterculic acid	294.47 g/mol	2	1	5.42	92.63	15	37.30 Å^2^	1
Tetradecanoic acid, methyl ester	242.40 g/mol	2	0	4.81	75.50	13	26.30 Å^2^	0

MW = molecular weight (g/mol); HBA = hydrogen bond acceptor; HBD = hydrogen bond donor; Log *p* = lipophilicity; AMR = molar refractivity; nRB = number of rotatable bond; TPSA = topological polar surface area.

**Table 5:** Toxicological property predictions of the selected compounds.

Compounds	Ames Toxicity	Carcinogens	Acute Oral Toxicity	Rat Acute Toxicity
1-azuleneethanol, acetate	AT	NC	III	1.8435
7,10-octadecadienoic acid, methyl ester	NAT	C	III	1.7357
9-octadecenoic acid (Z), methyl ester	NAT	NC	III	1.6684
Heptadecanoic acid, 14-methyl, methyl ester	NAT	C	III	1.5877
Hexadecanoic acid, methyl ester	NAT	C	III	1.4915
Sterculic acid	NAT	C	IV	1.5128
Tetradecanoic acid, methyl ester	NAT	C	III	1.4915

Here, NAT: Non AMES toxic; NC: Non Carcinogens; C: Carcinogens.

**Table 6:** Biological activities predicted for *S. foetida* major compounds by PASS online.

Compounds	Biological Activities Predicted by Pass Online	Pa	Pi
1-azuleneethanol, acetate	Anti-inflammatory Anti-hypoxic Antipyretic Antithrombotic Antinociceptive Anti-seborrheic	0.517 0.692 0.366 0.413 0.414 0.812	0.052 0.008 0.030 0.048 0.099 0.017
7, 10-octadecadienoic acid, methyl ester	Anti-inflammatory Anti-eczematic Anti-secretoric Antithrombotic Antinociceptive Antipyretic	0.728 0.953 0.781 0.706 0.593 0.255	0.013 0.002 0.005 0.008 0.008 0.068
9-octadecenoic acid (Z), methyl ester	Anti-inflammatory Hypolipemic Antipruritic Antithrombotic Antinociceptive Antipyretic	0.768 0.767 0.694 0.754 0.449 0.270	0.009 0.009 0.008 0.005 0.072 0.059
Heptadecanoic acid, 14-methyl, methyl ester	Anti-inflammatory Anti-secretoric Antipruritic Antithrombotic Antinociceptive Acetylesterase inhibitor	0.444 0.736 0.558 0.629 0.487 0.790	0.075 0.007 0.023 0.013 0.044 0.005
Hexadecanoic acid, methyl ester	Anti-inflammatory Anti-hypoxic Antipruritic Antithrombotic Antinociceptive Antiparasitic	0.758 0.620 0.566 0.587 0.538 0.429	0.002 0.016 0.021 0.017 0.019 0.025
Sterculic acid	Anti-inflammatory Antipyretic Anti-secretoric Antithrombotic Antinociceptive Anti-eczematic	0.558 0.349 0.785 0.576 0.436 0.866	0.004 0.033 0.005 0.018 0.081 0.008
Tetradecanoic acid, methyl ester	Anti-inflammatory Anti-eczematic Anti-seborrheic Antithrombotic Antinociceptive Antipyretic	0.728 0.854 0.777 0.706 0.593 0.323	0.013 0.009 0.002 0.008 0.008 0.038

## Data Availability

Available data are presented in the manuscript.

## Supplementary Materials

The following are available online at https://www.mdpi.com/article/10.3390/plants10061135/s1.
