# Supplementary material for: Chemical Profiling, Pharmacological Insights and In Silico Studies of Methanol Seed Extract of Sterculia foetida
Source: Plants (Basel). 2021 Jun 3;10(6):1135. doi: 10.3390/plants10061135 (PMC8227630; doi:10.3390/plants10061135)
Supplement: Supplementary file 1 [file plants-10-01135-s001.zip › plants-1215029-suppl.pdf]

Supplementary Materials

Article

Chemical Profiling, Pharmacological Insights and In Silico Studies of Methanol Seeds Extract of *Sterculia foetida*

Najmul Alam [1,2,†], Naureen Banu [1,†], Md. Arfin Ibn Aziz [1,2], Niloy Barua [1,2], Umme Ruman [1], Israt Jahan [1], Farhana Jahan Chy [1], Sushmita Debnath [3], Arkajyoti Paul [1,2], Md. Nazim Uddin Chy [1,2], Mohammed Aktar Sayeed [1,*], Talha Bin Emran [4,*] and Jesus Simal-Gandara [5,*]

1 Department of Pharmacy, International Islamic University Chittagong, Chittagong 4318, Bangladesh; nazmul9alam@gmail.com (N.A.); naureen2021@gmail.com (N.B.); arfinibnaziz151085@gmail.com (M.A.I.A); niloybaruaniloy@gmail.com (N.B.); umme.ruman.547@gmail.com (U.R.); istiisrat@gmail.com (I.J.); jahanfarhana47@gmail.com (F.J.C); nazim107282@gmail.com (M.N.U.C.)

2 Drug Discovery, GUSTO A Research Group, Chittagong 4203, Bangladesh

3 Comilla Medical College, Faculty of Medicine, University of Chittagong, Chittagong 4331, Bangladesh; sushmitaswarna@gmail.com (S.D.)

4 Department of Pharmacy, BGC Trust University Bangladesh, Chittagong 4381, Bangladesh; arka.bgctub@gmail.com (A.P.)

5 Nutrition and Bromatology Group, Department of Analytical and Food Chemistry, Faculty of Food Science and Technology, University of Vigo–Ourense Campus, E32004 Ourense, Spain

* Correspondence: jsimal@uvigo.es (J.S.G.); talhabmb@bgctub.ac.bd (T.B.E.); sayeed_ustc@yahoo.com (M.A.S.);
Tel.: +88-01819-942214 (T.B.E.); +34-988-387000 (J.S.G.)

Table S1. Phytochemical screening of methanol extract of *Sterculia foetida* seeds.

Test	Methanol Extract
Alkaloids	++
Glycoside	-
Quinones	-
Flavonoids	++
Saponins	+
Cholesterol	++
Carbohydrates	++
Phenol	+
Terpenoids	++
Steroids	+
Protein	+

Here, (+) weak presence; (++) presence; (−) absence.

Table S2. LC_{50} values with regression equation for MESF with reference to vincristine sulfate.

**LC_{50} Values (μg/mL)**		
**Chemicals/Plant Extracts**	**LC_{50}**	**Regression Equation**
**Vincristine Sulfate**	2.52 μg/ml	$y = 5.8247x + 35.285$; $R^2 = 0.928$
**MESF**	327.85 μg/ml	$y = 0.061x + 30.001$; $R^2 = 0.9817$

Here, MESF: methanol extract of *Sterculia foetida* seeds.
